# GWAS Links New Variant in Long Non-Coding RNA *LINC02006* with Colorectal Cancer Susceptibility

**DOI:** 10.3390/biology10060465

**Published:** 2021-05-25

**Authors:** Ewa E. Hennig, Anna Kluska, Magdalena Piątkowska, Maria Kulecka, Aneta Bałabas, Natalia Zeber-Lubecka, Krzysztof Goryca, Filip Ambrożkiewicz, Jakub Karczmarski, Tomasz Olesiński, Łukasz Zyskowski, Jerzy Ostrowski

**Affiliations:** 1 Department of Gastroenterology, Hepatology and Clinical Oncology, Centre of Postgraduate Medical Education, 02-781 Warsaw, Poland; mkulecka@cmkp.edu.pl (M.K.); natalia.zeber-lubecka@cmkp.edu.pl (N.Z.-L.); jostrow@warman.com.pl (J.O.); 2 Department of Genetics, Maria Skłodowska-Curie National Research Institute of Oncology, 02-781 Warsaw, Poland; anna.kluska@pib-nio.pl (A.K.); magdalena.piatkowska@pib-nio.pl (M.P.); aneta.balabas@pib-nio.pl (A.B.); kgoryca@gmail.com (K.G.); filip.ambrozkiewicz@pib-nio.pl (F.A.); jakub.karczmarski@pib-nio.pl (J.K.); 3 Department of Gastroenterological Oncology, Maria Skłodowska-Curie National Research Institute of Oncology, 02-781 Warsaw, Poland; tomasz.olesinski@pib-nio.pl (T.O.); lukasz.zyskowski@pib-nio.pl (Ł.Z.)

**Keywords:** colorectal cancer, genome-wide association study, tumor progression, metastasis, long non-coding RNA, polymorphism

## Abstract

**Simple Summary:**

Identifying risk factors for cancer development can allow for appropriate stratification and surveillance of individuals at risk, increasing their chances of benefiting from early disease detection; however, most of the genetic factors contributing to the risk of colorectal cancer (CRC) remain undetermined. Here, we adopted a new approach for selecting index polymorphism for further validation in combination with a genome-wide association study of pooled DNA samples for CRC susceptibility variants in the Polish population. This study, including 2013 patients and controls, uncovered five susceptibility loci not previously reported for CRC. Four of identified variants were located within genes likely involved in tumor invasiveness and metastasis, suggesting that they could be markers of poor prognosis in CRC patients. Our results provide evidence that conducting association studies on small but homogenous populations can help us discover new common risk variants specific to the studied population.

**Abstract:**

Despite great efforts, most of the genetic factors contributing to the risk of colorectal cancer (CRC) remain undetermined. Including small but homogenous populations in genome-wide association studies (GWAS) can help us discover new common risk variants specific to the studied population. In this study, including 465 CRC patients and 1548 controls, a pooled DNA samples-based GWAS was conducted in search of genetic variants associated with CRC in a Polish population. Combined with a new method of selecting single-nucleotide polymorphisms (SNPs) for verification in individual DNA samples, this approach allowed the detection of five new susceptibility loci not previously reported for CRC. The discovered loci were found to explain 10% of the overall risk of developing CRC. The strongest association was observed for rs10935945 in long non-coding RNA *LINC02006* (3q25.2). Three other SNPs were also located within genes (rs17575184 in *NEGR1,* rs11060839 in *PIWIL1,* rs12935896 in *BCAS3*), while one was intergenic (rs9927668 at 16p13.2). An expression quantitative trait locus (eQTL) bioinformatic analysis suggested that these polymorphisms may affect transcription factor binding sites. In conclusion, four of the identified variants were located within genes likely involved in tumor invasiveness and metastasis. Therefore, they could possibly be markers of poor prognosis in CRC patients.

## 1. Introduction

Colorectal cancer (CRC) is the third most commonly diagnosed malignant tumor, both around the world and in Poland [1]. It also represents the second and third leading cause of cancer-related deaths among men and women in the Polish population, respectively [2]. Genetic factors are thought to account for up to 35% of the variation in CRC risk [3,4]. Rare mutations with high penetration are responsible for less than 6% of cases of CRC [5,6]. In order to explain some of the remaining risk that contribute to CRC, numerous genome-wide association studies (GWAS) have been conducted for common low-penetrance variants. Currently, at least 100 independent susceptibility loci associated with CRC development at *p* < 5 × 10^−8^ have been identified, including over 50 new loci discovered in large-scale GWAS meta analyses in 2019 alone [7,8,9,10,11,12]. Despite these great efforts, less than 12% of familial relative risk [11] and less than 1% of the heritability of CRC [13] can be explained by the common variants identified by GWAS. Thus, most of the genetic factors contributing to the risk of CRC remain undetermined.

Given the significant population diversity in terms of genetic variation, and thus differences in allele frequencies and association strength, conducting analyses on different populations increases the chance of identifying general risk variants [14]. In addition, including ethnic or racial minorities can help to discover new loci or risk variants specific to the studied populations [15]. Our previous studies indicated that there are some benefits of studying relatively small but homogenous populations, such as the Polish population [16,17].

In several studies from recent years, we successfully implemented a novel approach for selecting GWAS-discovered single-nucleotide polymorphisms (SNPs) for further validation of their association with the disease [17,18,19,20]. This was shown to be effective particularly for GWAS with pooled DNA or with a small sample size, where limited study power hardly allows associations to be made at the standard genome-wide significance level (*p* < 5 × 10^−8^). In this approach, index SNPs for individual genotyping are selected based more on biological context than a purely statistical criterion, assuming that each associating SNP is usually not independent of neighboring variants. Such a method reduces the number of false-positive genome-wide associations and allows for the discovery of new associations [18]. Here, we adopted this method of selecting the index SNP in combination with pooled DNA sample GWAS for CRC susceptibility variants in the Polish population. This approach enabled the detection of five new susceptibility variants which have not yet been associated with CRC. Of these, four are intron variants of genes that are involved or very likely to be involved in the neoplastic process, especially tumor progression and metastasis. Interestingly, the strongest association was observed for the novel long non-coding RNA (lncRNA) variant, suggesting its role through interaction with transcription factors (TFs).

## 2. Materials and Methods

### 2.1. Ethics Statement

All patients and control subjects were Polish Caucasians recruited from two urban populations, Warsaw and Szczecin. The local ethics committee approved the study (Maria Skłodowska-Curie National Research Institute of Oncology, Warsaw, Poland, project ID: 37/2017/1/2021), and all subjects provided informed consent before they participated in the study. The study protocol conformed to the ethical guidelines of the 1975 Declaration of Helsinki.

### 2.2. Patients

In total, the pooled DNA sample-based GWAS and verification stage with individual samples included 2013 individuals—465 with CRC and 1548 controls. The pooled-sample GWAS cohorts included 432 patients with CRC (168 females and 264 males; median age: 66 years; range: 20–91 years) and 672 control subjects (360 females and 312 males; median age: 55 years; range: 19–95 years). The demographic and clinical characteristics of all patients and controls are shown in Table 1.

### 2.3. Genome-Wide Microarray Allelotyping

A pooled DNA sample-based GWAS was performed as described previously [21]. Genomic DNA was extracted from whole blood treated with EDTA using a QIAamp DNA Blood Mini Kit (Qiagen, Hilden, Germany), quantified using a Quant-iT^TM^ PicoGreen dsDNA Kit (Invitrogen, Carlsbad, CA, USA), and visually checked for integrity on 1% agarose gel. Solely DNA samples that passed quality control tests (for purity, quantity, and integrity) were combined according to diagnosis and gender at equimolar concentrations to obtain 24-sample pools. A total of 18 DNA pools were prepared for the CRC group (seven for women and 11 for men) and 28 for controls (15 for women and 13 for men). Pooled DNA samples were adjusted to a final concentration of 50 ng/L in Tris-EDTA buffer (pH = 8) and analyzed individually on Illumina Infinium Omni2.5-Exome-8 v1.3 BeadChip microarrays by a commercial organization (Eurofins Genomics, Galten, Denmark). The datasets from GWAS are available from the Gene Expression Omnibus (GEO) database under accession number GSE156411.

### 2.4. Individual Genotyping

According to our previously described approach [17,18,19,20] for the verification of GWAS findings, loci were chosen that were represented by blocks of SNPs associated with CRC at the *p* < 5 × 10^−3^, for which the intervals between all pairs of adjacent SNPs were <30 kb. From each of the independent loci, the most strongly associated SNP (at *p* < 10^−4^) was selected as an index SNP for further verification via individual DNA sample genotyping, and stepwise forward logistic regression analysis. TaqMan SNP Genotyping Assays (Thermo Fisher Scientific, Waltham, MA, U.S., a SensiMix™ II Probe Kit (Bioline Ltd., London, United Kingdom), and a 7900HT Real-Time PCR system (Thermo Fisher Scientific, U.S.) were used for individual genotyping in a 384-well format.

### 2.5. eQTL Analysis

Data collected in an online bioinformatics database by HaploReg, version 4.1 [22], was used for the analysis of identified CRC susceptibility genetic variants and the expression of quantitative trait loci (eQTL).

### 2.6. Survival Curves

Kaplan-Meier survival curves were prepared in Human Protein Atlas [23] (http://www.proteinatlas.org; accessed on 5 May 2021) using the default cut-off for differentiation between low and high gene expression and data from The Cancer Genome Atlas (TCGA) for the CRC patient cohort. *P*-values in the log-rank test were computed using default observation periods.

### 2.7. Statistical Analyses

#### 2.7.1. Genome-Wide Allelotyping

First, the relative allele signal (RAS) for each SNP was calculated as A/(A + B), where A and B were signal intensities for A and B alleles (as defined by Illumina). The RAS was used as an approximation of the allele ratio. Student’s *t*-test (Welch variant) was used to compare allele ratios between groups. Due to a lack of the call-rate statistics for pooled samples, the quality was assessed via visual inspection of the first two principal components for outliers (Figure S1). One control and one CRC pool were removed. The calculated lambda value of 1.007, together with the quantile–quantile (Q–Q) plot of *p*-values (Figure S2), raised no concerns regarding the homogeneity of the final population. No probe filtering was performed. *P*-values were corrected for multiple hypothesis testing with the Holm algorithm. Manhattan plotting was performed using the qqman R package [24]. Probe names were mapped to a reference SNP ID using mapping files (InfiniumOmni2-5-8v1-5 and InfiniumOmniExpressExome-8v1-6) provided by Illumina. The impact of variants on the coding sequence and clinical significance were imported with the biomaRt package [25,26]. All computations were performed according to the R environment [27]. The study power calculations were performed using the epiR package [28], assuming the proportion of an allele in a reference group of 0.05–0.5 and an odds ratio (OR) of 1.2–2 (Table S1). The method assumed a confidence level of 95% in an unmatched case–control study.

#### 2.7.2. Individual Genotyping

The Hardy–Weinberg equilibrium concordance of SNPs selected for verification was tested using the HardyWeinberg R package, version 1.6.8 [29], whereby no statistically significant deviations were observed. Differences in frequencies between groups were verified using the chi-squared test for alleles and the Cochran–Armitage test (implemented in R package DescTools, version 0.99.23) [30] for genotypes, with the exception of rs17575184, for which Fisher’s exact test was chosen due to the small number of alternative homozygotes. The *p*-value significance threshold was adjusted for multiple comparisons with the Benjamini–Hochberg algorithm [31]. The OR and 95% confidence interval (CI) were estimated by normal approximation implemented in the EpiTools R package, version 0.5–10 [32]. OR values were given, with the more frequent allele or genotype taken as the reference.

#### 2.7.3. Stepwise Forward Logistic Regression Analysis

Prediction analysis was performed by a stepwise forward logistic regression method, with the Akaike information criterion (AIC) used as the criterion for variable choice, using the step function of the R basic statistics package. The significant SNPs (*p* < 0.05) were ranked according to their AIC values, starting from a variant with the lowest AIC value, and sequentially introduced into the prediction model. Nagelkerke’s pseudo-*R*^2^ for each step was computed with the DescTools package, version 0.99.23 [30], in order to estimate the proportion of the overall risk of developing CRC. The area under the curve (AUC) value describing the accuracy of the prediction was computed using the pROC package, version 1.10 [33].

## 3. Results

### 3.1. Association Analyses

A pooled DNA sample-based GWAS in combination with a novel approach to SNP selection was applied in the search for new genetic variants associated with CRC in a Polish population. The 24-sample pools of DNA were used, obtained from 432 patients with CRC (18 pools) and 672 control subjects (28 pools). Seven independent loci were selected for further verification of their relationship to CRC development; neither showed statistically significant deviations from the Hardy–Weinberg equilibrium. Of these, six loci were represented by blocks of at least 11 SNPs associated with *p* < 5 × 10^−3^ at a distance of less than 30 kb from one another. One block (represented by rs17575184) consisted of seven SNPs, however three associated at *p* < 10^−4^. From each selected locus, the most strongly associated index SNP was further verified via genotyping of individual DNA samples from both the CRC (*N* = 465) and control (*N* = 1079) groups.

As shown in Table 2, in verification analyses, five of the GWAS-selected SNPs exhibit significant differences in allele and genotype frequencies between the CRC and control groups after the Benjamini–Hochberg algorithm’s adjustment for multiple testing (*p_adj_* < 0.05). None of these associations have previously been reported for CRC. The strongest association was observed for rs10935945 in *LINC02006* at 3q25.2 (*p_adj_* = 1.26 × 10^−5^ and 1.29 × 10^−5^ for allele and genotype frequencies, respectively). The next three SNPs revealed allelic associations at *p_adj_* ≤ 3.92 × 10^−4^. Apart from rs10935945, three other SNPs were located within gene regions (rs17575184 in *NEGR1* at 1p31.1, rs11060839 in *PIWIL1* at 12q24.33, and rs12935896 in *BCAS3* at 17q23.2), while one was at an intergenic location (16p13.2).

Additional association analyses, including stratifying the CRC patients cohort by tumor localization or different disease parameters (e.g., grading, metastasis), indicated that all five identified variants were significantly associated (*p_adj_* < 0.05) with G2 and T3 CRCs (Table S2). Interestingly, some significant associations were observed even when the CRC subgroup was very small, e.g., rs11060839 with G3 (*N* = 42) or rs10935945 with T4 (*N* = 56) and N2 (*N* = 78).

The minor allele (MA) of two SNPs was associated with an increased risk of CRC development, while that of the remaining three SNPs showed a protective effect (Table 2). The effect size of all five susceptibility loci was relatively moderate (OR ≥ 1.45 or ≤ 0.77), which is consistent with the estimated statistical power of our GWAS. Assuming an allele frequency of 0.3 to 0.5, a power ranging from 88% to 90% is needed to detect an effect size of OR = 1.5 (Table S1). The strongest effect was observed for rs17575184, located in the intron sequence of the *NEGR1* gene (OR = 0.57, 95% CI 0.42-0.76, *p_adj_* = 3.54 × 10^−4^).

### 3.2. Risk Prediction Modeling

To evaluate the contribution of individual SNPs to the risk of developing CRC, a stepwise forward logistic regression was performed with AIC minimization as a selection criterion. SNPs significant in the stepwise logistic regression were ranked according to their AIC value and sequentially introduced into the prediction model. Out of the seven SNPs selected for verification, only rs12424924 was excluded from further modeling (*p* > 0.05). According to the AIC estimates, the optimal model included six SNPs: rs9927668, rs10935945, rs17575184, rs12935896, rs11060839, and rs10838094 (Table 3). SNP rs9927668 emerged as the model with the lowest AIC value of all single-SNP models, and the addition of rs10935945 resulted in the largest AIC decrease. Both of these SNPs account for more than half of the risk of CRC development explained by the final model involving six SNPs. The sequential introduction of rs17575184, rs12935896, and rs11060839 moderately improved the parameters of the resulting models, while the inclusion of rs10838094 only marginally lowered the AIC. In total, the six SNPs included in the model were found to explain 10% of the overall risk of CRC development, as assessed using the Nagelkerke pseudo-*R^2^* statistic (Table 3). However, the overall accuracy expressed by an AUC value of 0.66 suggests the rather low predictability of the final model.

### 3.3. eQTL Bioinformatic Analysis

**Figure 1:**
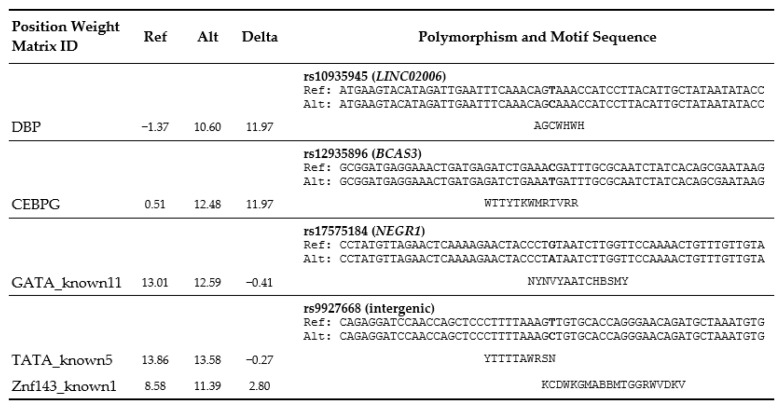
Possibly altered regulatory motifs. Based on HaploReg database (https://pubs.broadinstitute.org/mammals/haploreg/haploreg.php) [22]. Position weight matrix motifs in biological sequences [34]. Allele variants are indicated in bold. Ref, reference sequence; Alt, alternative sequence.

A search of the HaploReg database [22,34] revealed that four of the five SNPs significantly associated with CRC risk potentially changed TF binding motifs, which may implicate the regulatory effect of a variant (Figure 1). Among others, the predicted binding motifs of D-box binding PAR BZIP transcription factor (DBP) and CCAAT/enhancer-binding protein gamma (CEBPG) TFs overlap with the position of rs10935945 and rs12935896 polymorphisms, respectively.

### 3.4. Survival Probability

The expression levels of *PIWIL1, NEGR1,* and *BCAS3* did not significantly affect the probability of survival when analyzed in the full cohort of CRC patients and controls (*p* > 0.05; Figure S3). However, after stratification by gender, high relative *PIWIL1* expression at diagnosis was associated with a significantly lower survival probability in the male cohort (*p* = 0.02, 46% vs. 77% 5-year survival probability; Figure S3B), while low expression of *NEGR1* was correlated with a significantly lower 5-year survival probability among women (*p* = 0.049, 54% vs. 65%; Figure S3D).

## 4. Discussion

An important issue in GWAS is the large number of false positive results, to some extent due to the structure of the studied population, but also due to certain methodological assumptions. Defining an appropriate *p*-value threshold for statistical significance appears to be critical [35,36]. Our previous study revealed that most associations selected solely on the basis of the arbitrarily established genome-wide significance level (*p* < 5 × 10^−8^) turned out to be false positives, whereas the inclusion of biological context in the SNP selection method (by taking into account the strong allele linkage disequilibrium (LG)) significantly reduced the number of false positives [18]. This approach also increases the chance of finding new associations in small-sized studies and GWAS based on pooled DNA samples when the ability to reach the standard genome-wide significance level is limited [17,19]. The adoption of this method of selecting the index SNP for verification in combination with pooled DNA sample-based GWAS enabled the detection of five new genetic variants associated with CRC development in the Polish population. None of these SNPs have previously been reported to be associated with CRC. It cannot be determined whether the finding of new susceptibility loci resulted from the adopted methodological approach or because these variants are more specific to our population. It should be noted, however, that in the group of our study subjects, both clearly higher and lower frequencies of the MA of the identified variants were observed compared to those reported in the NCBI SNP database (Table 2), e.g., SNPs rs9927668 (0.391 vs. 0.290) and rs12935896 (0.252 vs. 0.400), respectively.

The strongest effect on CRC susceptibility was observed for SNP rs17575184, located within an intron of the neuronal growth regulator 1 (*NEGR1*) gene (OR = 0.57; Table 2). Previous GWASs have indicated an association of the rs17575184 polymorphism with asthma in children (*p* = 4 × 10^−3^) [37], whereas other SNPs in *NEGR1* were implicated in body weight regulation [38,39] and dyslexia [40]. NEGR1 is an extracellular adhesion protein that binds to cell membrane rafts, especially in the cell junction area, where it promotes cell-to-cell attachment and aggregation [41]. Given that adhesion properties are crucial in tumor cell migration and invasion during metastasis, NEGR1 may play a role in malignant transformation by regulating intercellular and cell-to-matrix interactions [42]. Accordingly, *NEGR1* was identified as a commonly downregulated gene in various types of human cancers, including CRC, suggesting its contribution to tumor suppression [41], while *NEGR1* overexpression reduced the tumorigenic properties of ovarian cancer cell line SKOV-3 cells [41]. When analyzed in the TCGA female CRC patients cohort, low expression of *NEGR1* was associated with the shorter 5-year survival rate (Figure S3D). NEGR1 may play a role in regulating neurite outgrowth and neuronal arborization via direct interaction with receptor tyrosine kinase fibroblast growth factor receptor 2 (FGFR2) [43,44]. *FGFR2* silencing inhibited cell migration and invasion [45], and its overexpression negatively correlated with overall CRC patient survival [46]. Therefore, NEGR1 may be functionally related to CRC, although the variant identified by GWAS is unlikely to be directly causal.

Similar to *NEGR1*, the breast-carcinoma-amplified sequence 3 (*BCAS3*) gene encoded a protein involved in cell adhesion and migration processes. The rs12935896, located in the intron sequence of *BCAS3,* was also associated with decreased CRC susceptibility (OR = 0.77; Table 2). *BCAS3* polymorphisms were previously associated via GWAS with gout [47], traits of kidney disease [48], and coronary artery disease [49]. Its misexpression was found in various types of cancer [50,51], and was implicated in tumor progression to a higher grade of malignancy [52]. BCAS3 is a cytoskeletal WD repeat domain-containing protein essential for angiogenesis, both during the developmental process and in tumor metastasis [51,53]. By activating and recruiting cell division cycle 42 (CDC42) Rho-GTPase [54] and facilitating crosstalk between cytoskeleton elements, BCAS3 regulates cell polarity and focal adhesion assembly [55].

SNP rs11060839 located in the intronic sequence of PIWI-like, RNA-mediated gene silencing 1 (*PIWIL1*) was associated with an increased risk of developing CRC (OR = 1.45; Table 2). PIWIL1 is a member of the PIWI-like family of Argonaute proteins, commonly associated with stem cell differentiation and self-renewal, RNA silencing, and the regulation of gene expression, whose activity is mediated by interactions with a specific class of small non-coding RNAs, referred to as PIWI-interacting RNAs (piRNAs) [56,57]. Both *PIWIL1* and piRNAs are overexpressed in CRC [58], and an upward trend was observed in *PIWIL1* expression levels during the colon adenoma–carcinoma sequence [59].

In patients with CRC, *PIWIL1* expression levels were closely related to the degree of tumor differentiation, TNM stage, the occurrence of lymph node invasion, and distant metastasis [59,60,61], suggesting that increased *PIWIL1* expression may promote tumor invasion. Moreover, patients with *PIWIL1* overexpression exhibited worse overall survival and disease-free survival, especially in the case of CRC at early stages or without lymph node invasion, showing the potential prognostic value of the *PIWIL1* expression status [61,62,63]. Accordingly, high expression levels of *PIWIL1* were associated with significantly lower 5-year survival probability when analyzed in the TCGA male CRC cohort (Figure S3B). Recently, a functional analysis of transcripts interacting with the PIWIL1–piRNA complex in the CRC COLO 205 cell line suggested that this complex may be directly involved in the activity regulation of key components of signal transduction cascades that are frequently dysregulated in CRC progression, including tumor suppressors and genes involved in the control of cell proliferation and differentiation, such as *IGF1R*, *JUN,* and *ERBB3* [64].

The strongest association reported in the current study was observed for rs10935945 in the lncRNA coding gene *LINC02006* at 3q25.2, which was associated with increased CRC risk. Although incapable of encoding proteins, lncRNAs play critical roles in the regulation of various cellular processes, such as cell growth, proliferation, apoptosis, and cancer progression [65]. LncRNAs can regulate gene expression, mainly at the post-transcriptional level, via various modes of direct action or as miRNA sponges or endogenous competitors, thus reducing their regulatory effect on target mRNAs [66]. The aberrant expression of lncRNAs, exemplified by *lncRNA H19* and *lncRNA 91H*, an antisense gene of H19, has been implicated in the tumorigenesis and metastasis of different types of cancer, including CRC, where it is associated with a poor prognosis and a high risk of tumor metastasis [67,68]. Both *LINC01354* and *lncRNA CASC11* are upregulated in CRC and contribute to the proliferation, invasion, and metastasis of CRC via activation of the Wnt/β-catenin signaling pathway [69,70]. Additionally, *LINC01123* was upregulated in CRC tumors and cells, and its expression positively correlated with the vascular endothelial growth factor A (*VEGFA*) expression and the binding of miR-34c-5p, sponged by LINC01123 [71]. The silencing of *UNC5B-AS1,* highly expressed in CRC tissues, repressed cancer growth and metastasis, most likely by increasing miR-622 expression and suppression of the AMP-activated protein kinase (AMPK) and the phosphatidylinositol 3-kinase (PI3K)/protein kinase B (AKT) signaling pathways [72]. On the other hand, overexpression of *lncRNA TUSC7* reduces cell migration and invasion in CRC by sponging miR-211 [73].

Several genetic variants located in lncRNA genes influence the risk of CRC development; polymorphisms in *lncRNA HOTTIP,* rs145204276 and rs55829688 in *lncRNA GAS5,* rs2839698 in *lncRNA H19*, rs2632159 in *lncRNA PCAT1*, rs2147578 in *lnc-LAMC2-1:1,* and rs664589 in *MALAT1* were associated with a significantly increased CRC risk [67], while rs13252298 and rs1456315 in *lncRNA PRNCR1* and rs1194338 in *MALAT1* had protective effects on CRC [67,74]. It has been suggested that the rs664589 G allele alters the binding of MALAT1 to miR-194-5p, resulting in an increased expression of *MALAT1* and enhanced CRC development and metastasis [75], while the rs2147578 in *lnc-LAMC2-1:1* affects the sponging of miR-128-3p, which correlates with higher expression of the *LAMC2* oncogene in CRC [76]. The rs55829688 in the *lncRNA GAS5* promoter region exerts its regulatory effect by affecting the binding affinity of TF YY1 to the *GAS5* promoter and downregulating *GAS5* expression [77]. Additionally, the *lncRNA PCAT1* rs2632159 may impact the risk of CRC by modulating the binding of EBF, LUN-1, and TCF12 [78], which could stimulate *PCAT1* expression, thus increasing its oncogenic function. Similarly, an eQTL bioinformatic analysis showed that the rs10935945 T variant of *LINC02006* identified in this study could influence binding with the TF DBP, a member of the PAR leucine zipper TF family (Figure 1), possibly increasing the risk of CRC development.

Based on the above-mentioned functional relations to CRC, it can be speculated that the gene variants identified in this study may be associated with metastatic and invasive CRC. Accordingly, after stratification of the CRC patient cohort by different disease parameters, all five identified variants were significantly associated with rather advanced tumors (T3; Table S2). Moreover, when comparing the CRC subgroup with the metastases to the control group, associations of three variants (rs11060839, rs9927668, and rs12935896) were observed, although they did not remain significant after statistical adjustment. However, it should be borne in mind that the lack of certain associations could be due to the small size of the analyzed subgroups, and all these observations need to be validated in a bigger, independent study. Our study included CRC patients with relatively advanced neoplastic disease, as indicated by the clinical characteristics of the patients (Table 1). Apart from the differences resulting from the use of technologically different platforms, this may also be the reason why the associations of known GWAS variants did not reach statistical significance. Moreover, the specific genetic architecture of the Polish population may also be important, which is consistent with the results of the recent replication study in the Basque population [79].

## 5. Conclusions

In this study, five new susceptibility variants associated with CRC development were revealed by the pooled DNA sample GWAS in a Polish population. Among them, four are intron variants of genes encoding proteins that are likely involved in the neoplastic process, especially tumor invasiveness and metastasis, and therefore could possibly be markers of poor prognosis in CRC patients. In total, discovered loci were found to account for 10% of the variation in the risk of developing CRC. While the prediction accuracy of the built model was rather low, the newly identified variants can significantly improve the cumulative risk assessment of CRC based on common susceptibility variants.

In line with the growing body of data suggesting that SNPs in lncRNAs can influence CRC risk, the novel lncRNA variant *LINC02006* was shown to express the strongest association with CRC development, possibly by affecting the DBP TK binding site and deregulating downstream pathways. Further understanding of lncRNA functions in cancer progression could improve CRC prediction and diagnosis.

## Figures and Tables

**Table 1:** The demographic and clinical characteristics of patients and controls.

	CRC (*N* = 465)	Control (*N* = 1548)
Female *N* (%)	176 (38)	969 (63)
Male *N* (%)	289 (62)	579 (37)
Age (mean ± SD)	66 ± 11	55 ± 11
Age (median)	66	58
Age (min.–max.)	20–91	19–95
Tumor localization (%)		
rectum	173 (37.2)	
sigmoid	79 (17.0)	
sigmoid-rectum	72 (15.5)	
caecum	55 (11.8)	
ascendant	39 (8.4)	
other	47 (10.1)	
Tumor size (%)		
0	4 (0.9)	
1	40 (8.6)	
2	85 (18.3)	
3	277 (59.6)	
4	56 (12.0)	
Tis	3 (0.6)	
Node status (%)		
0	245 (52.7)	
1	126 (27.1)	
2	78 (16.8)	
3	9 (1.9)	
Nx	7 (1.5)	
Grade (%)		
1	27 (5.8)	
2	284 (61.1)	
3	44 (9.5)	
Gx	110 (23.6)	
Metastasis (%)	49 (10.5)	

CRC, colorectal cancer; *N*, number of subjects; SD, standard deviation; Tis, tumor in situ; Nx, indeterminate; Gx, indeterminate.

**Table 2:** The allelic and genotypic association of GWAS-selected, single-nucleotide polymorphisms (SNPs) with colorectal cancer.

		Allele Frequency (%)						Genotype Frequency (%)							
dbSNP ID ^a^	Region	MA	MAF ^b^	Control	CRC	OR (95% CI)	*p_adj_-*Value	Genotype	Control	CRC		OR (95% CI)		*p_adj_-*Value	
**rs17575184**	1p31.1 *NEGR1* intron	A	0.088	232 (10.8)	60 (6.5)	0.57 (0.42–0.76)	**3.54 × 10^−4^**	AA AG GG	11 (1.0) 210 (19.6) 852 (79.4)	1 (0.2) 58 (12.5) 406 (87.3)		0.22 (0.01–1.12) 0.58 (0.42–0.79) -		**7.91 × 10^−4^**	
**rs10935945**	3q25.2 *LINC02006* intron	T	0.399	906 (42.2)	478 (51.5)	1.46 (1.25–1.70)	**1.26 × 10^−5^**	TT TC CC	195 (18.2) 516 (48.0) 363 (33.8)	117 (25.2) 244 (52.6) 103 (22.2)		2.11 (1.54–2.90) 1.66 (1.28–2.18) -		**1.29 × 10^−5^**	
rs10838094	11p15.4 *OR51B5* intron	A	0.378	445 (41.4)	422 (45.5)	1.18 (0.99–1.41)	8.03 × 10^−^^2^	AA AG GG	97 (18.1) 251 (46.7) 189 (35.2)	92 (19.8) 238 (51.3) 134 (28.9)		1.34 (0.93–1.92) 1.34 (1.01–1.78) -		8.12 × 10^−^^2^	
rs12424924	12p12.1 *PYROXD1* intron	A	0.194	223 (20.5)	165 (17.9)	0.85 (0.68–1.06)	0.147	AA AG GG	25 (4.6) 173 (31.8) 346 (63.6)	16 (3.5) 133 (28.9) 311 (67.6)		0.71 (0.37–1.36) 0.86 (0.65–1.12) -		0.152	
**rs11060839**	12q24.33 *PIWIL1* intron	A	0.169	332 (15.6)	194 (21.1)	1.45 (1.19–1.76)	**3.92 × 10^−4^**	AA AG GG	27 (2.5) 278 (26.1) 760 (71.4)	21 (4.6) 152 (33.0) 287 (62.4)		2.06 (1.13–3.71) 1.45 (1.14–1.84) -		**5.70 × 10^−4^**	
**rs9927668**	16p13.2 intergenic -	C	0.290	840 (39.1)	285 (30.9)	0.70 (0.59–0.82)	**5.04 × 10^−5^**	CC CT TT	179 (16.7) 482 (44.9) 412 (38.4)	46 (10.0) 193 (41.9) 222 (48.2)		0.48 (0.33–0.68) 0.74 (0.59–0.94) -		**8.25 × 10^−5^**	
**rs12935896**	17q23.2 *BCAS3* intron	C	0.400	545 (25.4)	194 (20.9)	0.77 (0.64–0.93)	**8.85 × 10^−3^**	CC CT TT	68 (6.4) 409 (38.2) 594 (55.5)	21 (4.5) 152 (32.7) 292 (62.8)		0.63 (0.37–1.03) 0.76 (0.60–0.95) -		**8.87 × 10^−3^**	

Allelic frequencies of all studied SNPs were in Hardy–Weinberg equilibrium. Bold denotes significant association after Benjamini–Hochberg algorithm adjustment (*p_adj_* < 0.05). CRC, colorectal cancer; MA, minor allele; MAF, MA frequency; OR, odds ratio; CI, confidence interval. ^a/^ SNP identifier based on NCBI SNP database (http://www.ncbi.nlm.nih.gov/snp/; accessed on 20 November 2020). ^b/^ MAF based on NCBI SNP database (http://www.ncbi.nlm.nih.gov/snp/; accessed on 20 November 2020).

**Table 3:** The results of the stepwise selection for the logistic regression model.

dbSNP ID ^a^	AIC ^b^	AIC Change (%)	*R* ^2 c^	*R*^2^ Change (%)
rs9927668	1309.45		0.028	
rs10935945	1294.45	15.0 (1.15)	0.054	0.026 (92)
rs17575184	1285.31	9.14 (0.74)	0.071	0.017 (33)
rs12935896	1279.74	5.57 (0.43)	0.084	0.013 (18)
rs11060839	1274.75	4.99 (0.39)	0.095	0.012 (14)
rs10838094	1274.26	0.49 (0.04)	0.101	0.006 (6)

Six significant SNPs (*p* < 0.05) ranked by Akaike information criterion (AIC) values were sequentially implemented into the model, starting with SNP rs9927668 with the lowest AIC value. All six SNPs were included in the final prediction model. ^a/^ SNP identifier based on NCBI SNP database (http://www.ncbi.nlm.nih.gov/SNP/; accessed on 20 November 2020). ^b/^ AIC value calculated after sequential implementation of the ranked SNPs. ^c/^ Nagelkerke pseudo-R^2^ value calculated after sequential implementation of the ranked SNPs.

## Data Availability

The data supporting the findings of this study are available in this article. The datasets from GWAS are available at the Gene Expression Omnibus (GEO) database under accession number GSE156411.

## Supplementary Materials

The following are available online at https://www.mdpi.com/article/10.3390/biology10060465/s1: Figure S1: A plot of the first two principal components for the relative allele signal (RAS) values of all analyzed microarray samples. Figure S2: A quantile–quantile (Q–Q) plot of the 2,561,761 *t* statistics from the whole-exome microarray analysis of 18 CRC and 28 control pooled DNA samples. Figure S3: The Kaplan–Meier curves for the association of the expression levels of identified genes with survival probability in colorectal cancer patients, according to the *Human Protein Atlas* database. Table S1: The power of the study calculated for a given minor allele frequency (first column) and odds ratio (second row). Table S2: The significant allelic association of GWAS-selected SNPs with colorectal cancer parameters.
