# Supplementary material for: GWAS Links New Variant in Long Non-Coding RNA LINC02006 with Colorectal Cancer Susceptibility
Source: Biology (Basel). 2021 May 25;10(6):465. doi: 10.3390/biology10060465 (PMC8229782; doi:10.3390/biology10060465)
Supplement: Supplementary file 1 [file biology-10-00465-s001.zip › Figure S3.pdf]

Figure S3. The Kaplan-Meier curves for the association of the expression levels of identified genes with survival probability in colorectal cancer patients, according to the *Human Protein Atlas* database.

A. ***PIWIL1*** *P = 0.083*

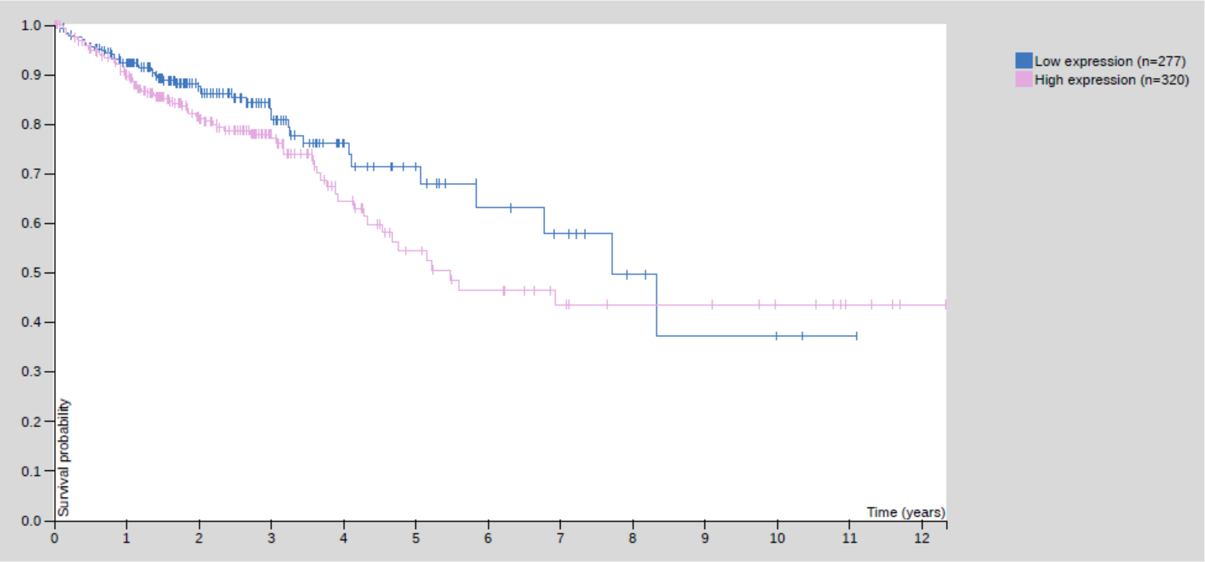

B. ***PIWIL1*** *(male)* *P = 0.02*

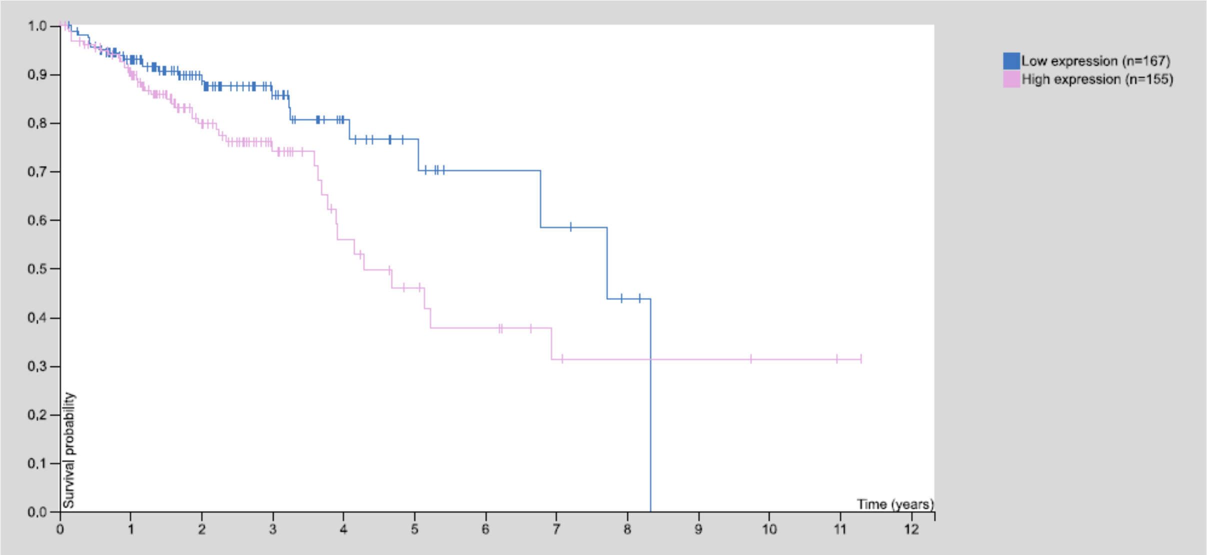

C. ***NEGR1*** *P = 0.12*

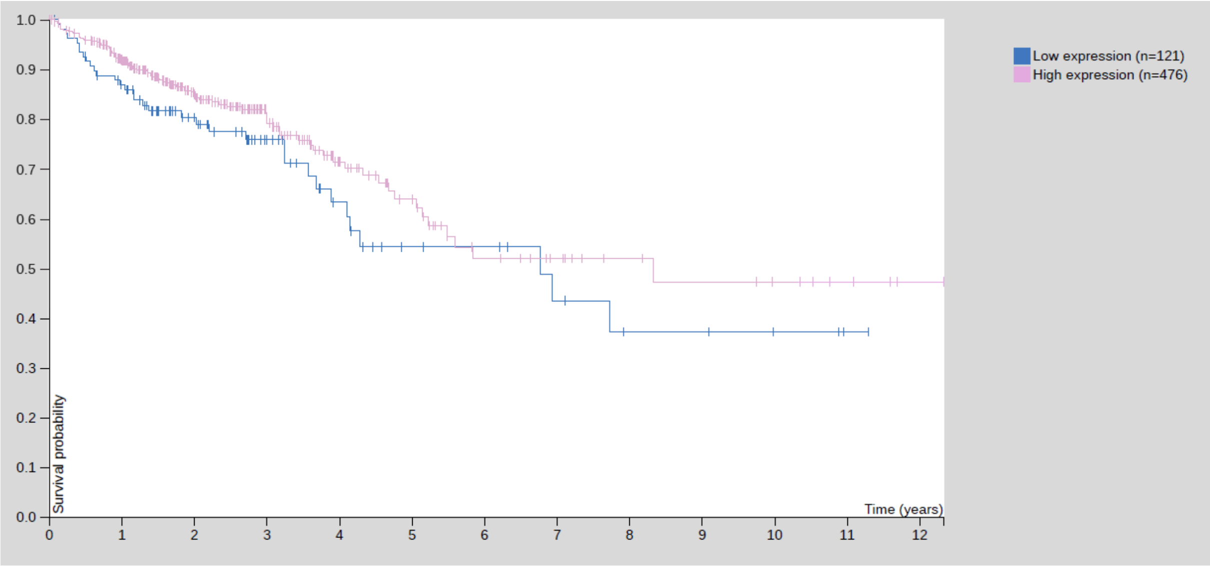

D. ***NEGR1*** *(female)* *P = 0.049*

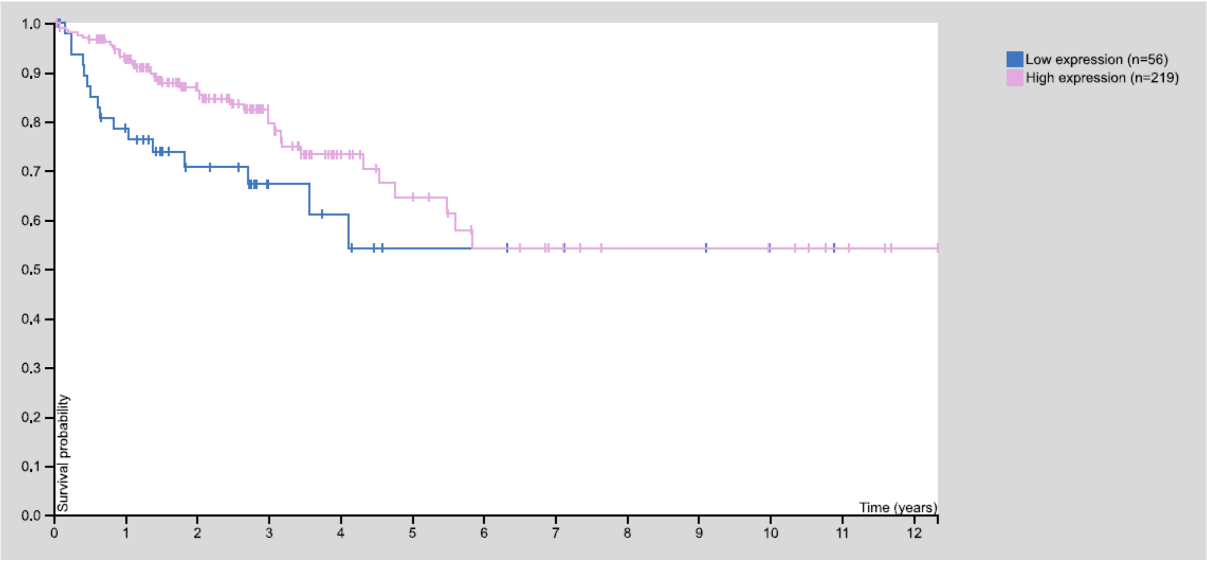

E. ***BCAS3*** *P = 0.12*

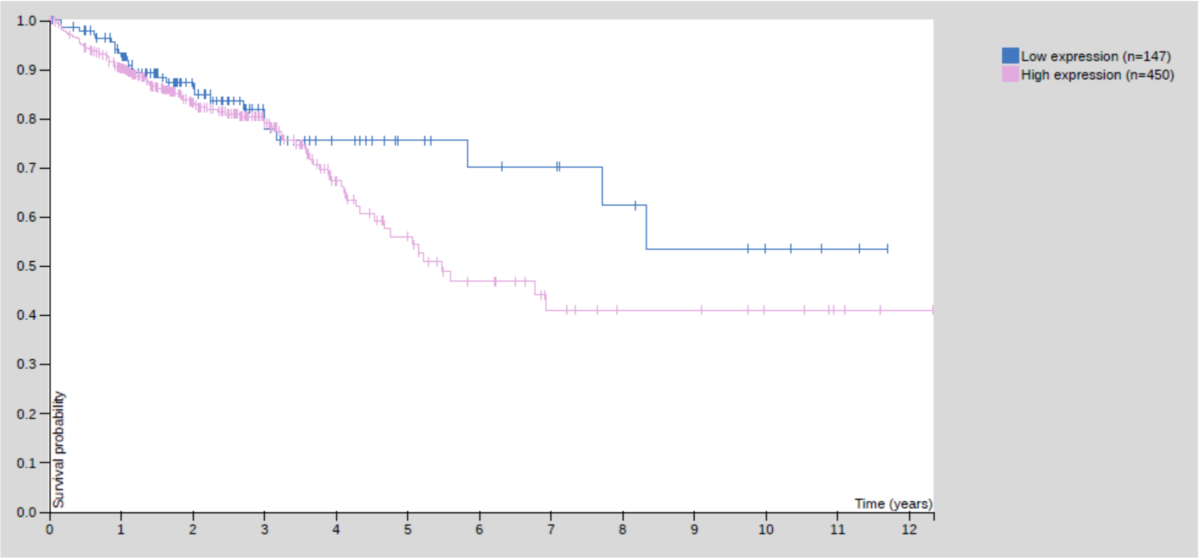

Low expression (n=147)
High expression (n=450)
Survival probability
Time (years)
1.0
0.9
0.8
0.7
0.6
0.5
0.4
0.3
0.2
0.1
0.0
0
1
2
3
4
5
6
7
8
9
10
11
12
