# Supplementary material for: GWAS Links New Variant in Long Non-Coding RNA LINC02006 with Colorectal Cancer Susceptibility
Source: Biology (Basel). 2021 May 25;10(6):465. doi: 10.3390/biology10060465 (PMC8229782; doi:10.3390/biology10060465)
Supplement: Supplementary file 1 [file biology-10-00465-s001.zip › Table S2.pdf]

Table S2. The significant allelic association of GWAS-selected SNPs with colorectal cancer parameters.

**Compared groups (*N*)**	**dbSNP ID** [a]	**Region**	**OR (95% CI)**	***p*-value**	**p_{adj}-value**
G2 (*284*) vs. control (*1079*)	rs17575184	*NEGR1*	0.59 (0.41-0.84)	4.53×10^{-3}	**7.55×10^{-3}**
	rs10935945	*LINC02006*	1.47 (1.22-1.77)	4.40×10^{-5}	**2.20×10^{-4}**
	rs11060839	*PIWIL1*	1.37 (1.08-1.73)	8.58×10^{-3}	**3.92×10^{-4}**
	rs9927668	intergenic	0.69 (0.56-0.84)	2.00×10^{-4}	**4.90×10^{-4}**
	rs12935896	*BCAS3*	0.78 (0.62-0.97)	3.00×10^{-2}	**3.00×10^{-2}**
G3 (*42*) vs. control (*1079*)	rs11060839	*PIWIL1*	2.06 (1.22-3.37)	4.34×10^{-3}	**2.17×10^{-2}**
	rs12935896	*BCAS3*	0.49 (0.25-0.88)	2.06×10^{-2}	5.14×10^{-2}
T3 (*277*) vs. control (*1079*)	rs17575184	*NEGR1*	0.63 (0.43-0.88)	8.45×10^{-3}	**1.06×10^{-2}**
	rs10935945	*LINC02006*	1.35 (1.12-1.63)	1.63×10^{-3}	**2.71×10^{-3}**
	rs11060839	*PIWIL1*	1.48 (1.16-1.87)	1.12×10^{-3}	**2.71×10^{-3}**
	rs9927668	intergenic	0.70 (0.57-0.85)	4.40×10^{-4}	**2.28×10^{-3}**
	rs12935896	*BCAS3*	0.77 (0.61-0.96)	2.22×10^{-2}	**2.22×10^{-2}**
T4 (*56*) vs. control (*1079*)	rs10935945	*LINC02006*	2.04 (1.39-3.02)	2.37×10^{-4}	**1.18×10^{-3}**
N0 (*245*) vs. control (*1079*)	rs10935945	*LINC02006*	1.46 (1.20-1.78)	1.42×10^{-4}	**7.10×10^{-4}**
	rs11060839	*PIWIL1*	1.36 (1.06-1.74)	1.56×10^{-2}	**2.42×10^{-2}**
	rs9927668	intergenic	0.75 (0.61-0.93)	7.79×10^{-3}	**1.95×10^{-2}**
	rs12935896	*BCAS3*	0.75 (0.59-0.95)	1.93×10^{-2}	**2.42×10^{-2}**
N2 (*78*) vs. control (*1079*)	rs10935945	*LINC02006*	1.69 (1.22-2.35)	1.63×10^{-3}	**8.13×10^{-3}**
	rs9927668	intergenic	0.68 (0.47-0.97)	3.37×10^{-2}	8.42×10^{-2}
RC (*173*) vs. control (*1079*)	rs17575184	*NEGR1*	0.48 (0.29-0.76)	2.28×10^{-3}	**5.70×10^{-3}**
	rs10935945	*LINC02006*	1.59 (1.27-2.00)	5.61×10^{-5}	**2.80×10^{-4}**
Metastasis (*49*) vs. control (*1079*)	rs11060839	*PIWIL1*	1.81 (1.10-2.88)	1.38×10^{-2}	5.30×10^{-2}
	rs9927668	intergenic	0.59 (0.37-0.92)	2.12×10^{-2}	5.30×10^{-2}
	rs12935896	*BCAS3*	0.53 (0.29-0.91)	3.18×10^{-2}	5.30×10^{-2}

Allelic frequencies of all studied SNPs were in Hardy-Weinberg equilibrium. Bold denotes significant association after Benjamini-Hochberg algorithm adjustment ($p_{adj} < 0.05$). RC; rectal cancer, OR; odds ratio, CI; confidence interval, G; grading, T; tumor size, N; lymph node status.
[a/] SNP identifier based on NCBI SNP database (*http://www.ncbi.nlm.nih.gov/snp/*).
