# Supplementary figures and images for: GWAS Links New Variant in Long Non-Coding RNA LINC02006 with Colorectal Cancer Susceptibility
Source: Biology (Basel). 2021 May 25;10(6):465. doi: 10.3390/biology10060465 (PMC8229782; doi:10.3390/biology10060465)

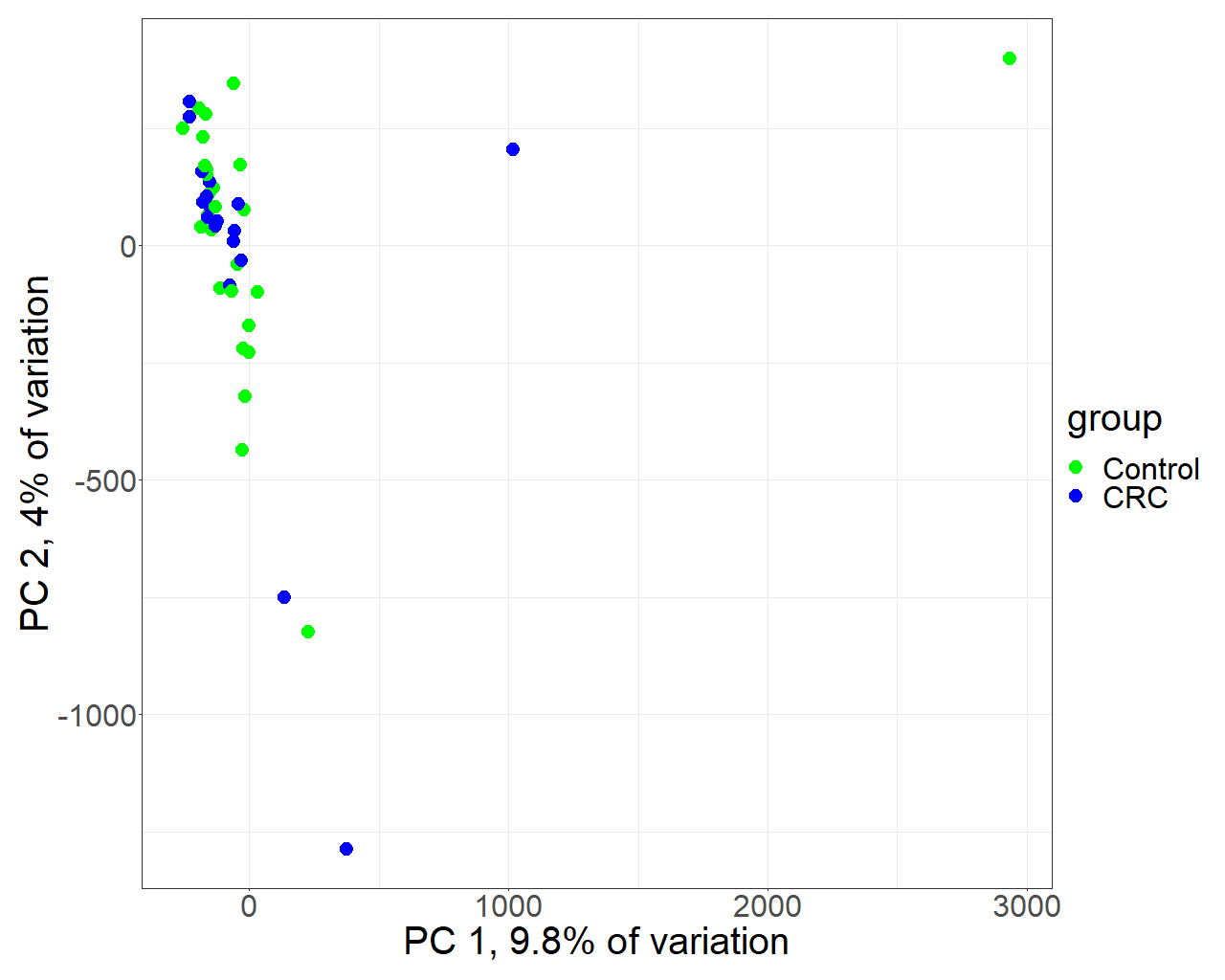

Supplement: Supplementary file 1 [file biology-10-00465-s001.zip › Figure S1.tiff]

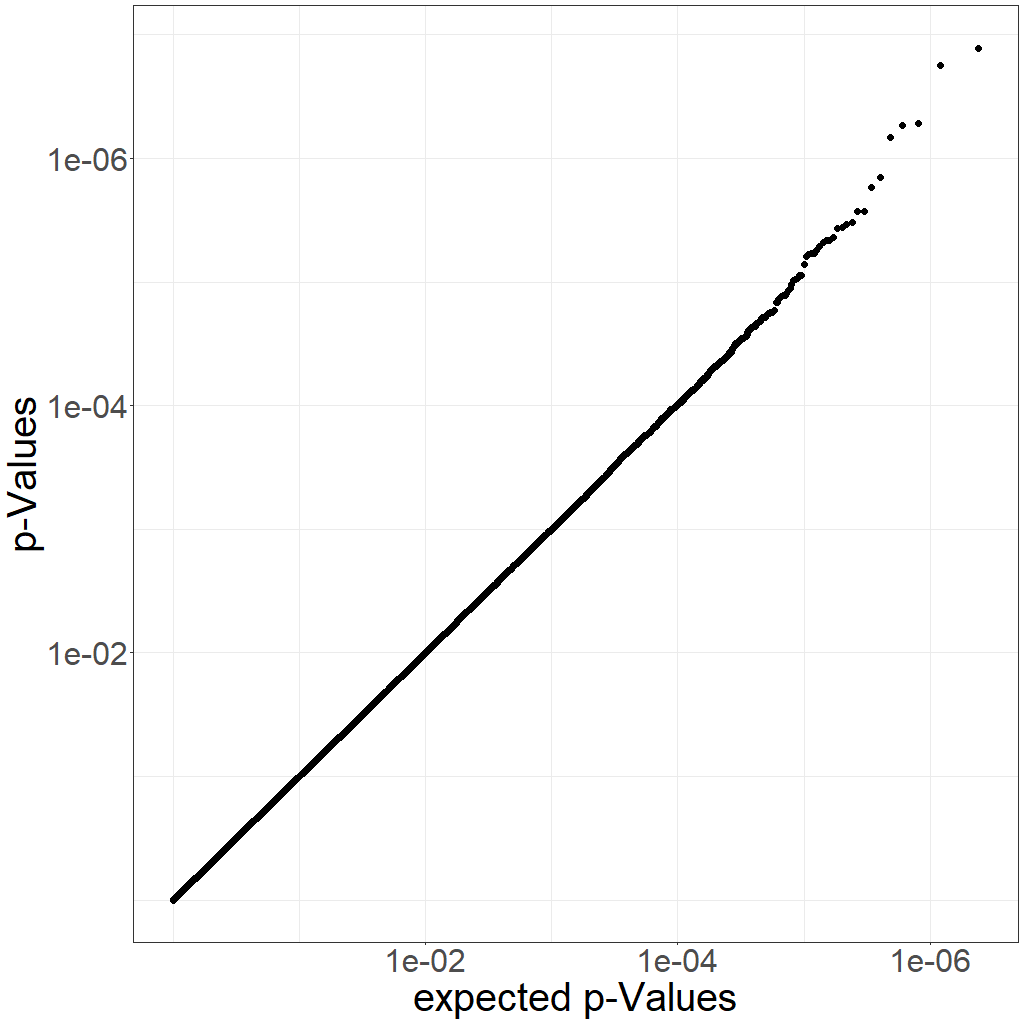

Supplement: Supplementary file 1 [file biology-10-00465-s001.zip › Figure S2.tiff]
